# Phenolic Profiles, Antioxidant, and Inhibitory Activities of *Kadsura heteroclita* (Roxb.) Craib and *Kadsura coccinea* (Lem.) A.C. Sm.

**DOI:** 10.3390/foods9091222

**Published:** 2020-09-02

**Authors:** Varittha Sritalahareuthai, Piya Temviriyanukul, Nattira On-nom, Somsri Charoenkiatkul, Uthaiwan Suttisansanee

**Affiliations:** 1 Institute of Nutrition, Mahidol University, Salaya, Phuttamonthon, Nakhon Pathom 73170, Thailand; varittha.sri@hotmail.com (V.S.); piya.tem@mahidol.ac.th (P.T.); nattira.onn@mahidol.ac.th (N.O.-n.); somsri.chr@mahidol.ac.th (S.C.); 2 Food and Nutrition Academic and Research Cluster, Institute of Nutrition, Mahidol University, Salaya, Phuttamonthon, Nakhon Pathom 73170, Thailand

**Keywords:** *Kadsura* spp., fruit parts, phenolics, antioxidant capacity, in vitro health properties

## Abstract

*Kadsura* spp. in the Schisandraceae family are woody vine plants, which produce edible red fruits that are rich in nutrients and antioxidant activities. Despite their valuable food applications, *Kadsura* spp. are only able to grow naturally in the forest, and reproduction handled by botanists is still in progress with a very low growth rate. Subsequently, *Kadsura* spp. were listed as endangered species by the International Union for Conservation of Nature and Natural Resources (IUCN) in 2011. Two different *Kadsura* spp., including *Kadsura coccinea* (Lem.) A.C. Sm. and *Kadsura heteroclita* (Roxb.) Craib, are mostly found in northern Thailand. These rare, wild fruits are unrecognizable to outsiders, and there have only been limited investigations into its biological properties. This study, therefore, aimed to comparatively investigate the phenolic profiles, antioxidant activities, and inhibitory activities against the key enzymes involved in diabetes (α-glucosidase and α-amylase) and Alzheimer’s disease (acetylcholinesterase (AChE), butyrylcholinesterase (BChE), and beta-secretase 1 (BACE-1)) in different fruit parts (exocarp, mesocarp (edible part), seed, and core) of *Kadsura coccinea* (Lem.) A.C. Sm. and *Kadsura heteroclita* (Roxb.) Craib. The results suggested that *Kadsura* spp. extracts were rich in flavonol (quercetin), flavanone (naringenin), anthocyanidins (cyanidin and delphinidin), and anthocyanins (cyanidin 3-*O*-glucoside (kuromanin), cyanidin 3-*O*-galactoside (ideain), cyanidin 3-*O*-rutinoside (keracyanin), and cyanidin 3,5-di-*O*-glucoside (cyanin)). These flavonoids were found to be responsible for the high antioxidant activities and key enzyme inhibitions detected in *Kadsura* spp. extracts. The findings of the present study can support further development of *Kadsura* spp. as a potential source of phenolics and anti-oxidative agents with health benefits against diabetes and Alzheimer’s disease. Besides, exocarp and the core of *Kadsura* spp. exhibited higher phenolic contents, antioxidant activities, and key enzyme inhibitory activities compared to the mesocarp and seeds, respectively. This information can promote the use of fruit parts other than the edible mesocarp for future food applications using *Kadsura* spp. rather than these being wasted.

## 1. Introduction

For thousands of years, humans have consumed naturally indigenous plants as green medicines to treat illnesses, including vegetables, herbs, and fruits. However, the development of effective, advanced scientific technology and synthetic drugs has significantly lowered the popularity of the natural medicine approach. Besides several advantages, including ready-to-use therapeutic applications and the commercial availability of synthetic drugs, severe side effects and economic impacts are other factors of concern. These issues have brought indigenous plants back into focus. Natural products have been considered as better functional food sources for alternative approaches in the prevention of certain non-communicable diseases (NCDs), and have even been used as green medicine to treat particular diseases. Subsequently, the study of medicinal applications and functional food development from beneficial plants are currently in focus.

*Kadsura* spp. in the *Schisandraceae* family produce ovate-elliptical shaped leaves and solitarily unisexual flowers [1]. The globose fruit is between 14 and 20 cm in diameter and forms a hexagonal structured skin developed by each carpel [1]. The fruit shape is similar to that of sugar-apples, but with a sourer and astringent taste. While the ripened fruit is edible [2], other plant parts can also be used as traditional medicine [1]. The stem and root of *Kadsura coccinea* are used to reduce rheumatic pain in the bones, chronic enteritis, acute gastritis, and the immunologic hepatic fibrosis effect [3], while those of *Kadsura longipedunculata* are used in the treatment of rheumatic arthritis, traumatic injury, dysmenorrhea, abdominal pain, irregular menstruation, canker sores, and gastrointestinal inflammation [3]. Moreover, the *Kadsura heteroclita* stem can prevent and treat rheumatic and arthritic diseases with anti-nociceptive and anti-inflammatory effects [4,5]. Phenolics detected in *Kadsura* spp. appear to be responsible for these health properties. Flavonoids identified in *Kadsura oblongifolia*, including quercetin and kaempferol, possess potent antioxidant activities [6]. Additionally, *K. coccinea* has been previously reported to contain gallic acid as a major phenolic acid, which has strongly demonstrated DPPH-radical scavenging activity (the half maximal inhibitory concentration (IC_50_) = 23.64 µg/mL) compared to commercial antioxidants, ascorbic acid, and 2,6-ditertbuty l-4-methylphenol (BHT) (the IC_50_ of 48.46 and 975.96 µg/mL, respectively) [7]. However, stressful conditions i.e., aphid feeding [8], light and drought intensity [9,10], or even the processing of biological material [11] can significantly affect the antioxidant properties or the phenolic profile.

The local Thai name for *Kadsura* spp. is noi-na-kreau and was first discovered in northern Thailand in 1972 [12]. *Kadsura* spp. were listed as an endangered species by the International Union for Conservation of Nature and Natural Resources (IUCN) in 2011, and since then only 11 trees have been found in the Thai provinces of Chiang Rai, Chiang Mai, and Mae Hong Son. Due to how rare they are, *Kadsura* spp. were listed by the Plant Genetic Conservation Project under the initiative of Her Royal Highness Princess Maha Chakri Sirindhorn (RSPG) in the same year of 2011, aiming to sustainably conserve and allocate plant resources to optimize beneficial utilizations [13]. Unlike more ubiquitous fruits in Thailand, *Kadsura* spp. are not well known, and due to largely being unrecognized, they are at high risk of extinction. In order to obtain the main goal of RSPG to sustainably conserve *Kadsura* spp. by expanding the knowledge on their biological properties, eventually leading to proper management of the plant, the objective of the present study is, therefore, to examine the phenolic profiles, antioxidant activities, and in vitro key enzyme inhibitory properties of *K. coccinea* (Lem.) A.C. Sm. and *K. heteroclita* (Roxb.) Craib, the two most abundant *Kadsura* spp. found in northern Thailand. These enzyme inhibitory properties are related to medicinal abilities against diabetes and Alzheimer’s disease (AD) by inhibiting key enzymes, which control these diseases. Targeting the inhibition of the carbohydrate degrading enzymes, including α-glucosidase and α-amylase, is a key approach for drugs designed to control diabetes. Based on enzyme–inhibitor structural interactions, several phenolics have been proven as effective α-glucosidase and α-amylase inhibitors [5,14]. In addition, neurotransmitter degrading enzymes, acetylcholinesterase (AChE) and butyrylcholinesterase (BChE), and amyloid precursor protein (APP) degrading enzyme, beta-secretase (BACE-1), are the key enzymes, which control the occurrence of AD. It has been previously suggested that the extreme loss of cholinergic markers found in the cerebral cortex is associated with AD, while BACE-1 causes the formation of beta-amyloid peptides or senile plaques, a hallmark of AD [15]. The findings of the present study provide valuable information for the sustainable conservation of the nearly extinct *Kadsura* spp. by describing their advantageous biological properties and potential applications, which can help to establish proper management and prolong the existence of the plants.

## 2. Materials and Methods

### 2.1. Sample Collection, Preparation, and Extraction

*Kadsura coccinea* (Lem.) A.C. Sm. and *Kadsura heteroclita* (Roxb.) Craib were collected from forests near Hui-Nam-Guen village, Mae-Jedi-Mai sub-district, Weing-Pa-Pao district, Chiang Rai Province, Thailand (19°11′58.3″ N and 99°31′00.7″ E). The fruits of both species could be harvested only once a year at different time periods. The fruits of *Kadsura coccinea* (Lem.) A.C. Sm. were collected in October 2017, while those of *Kadsura heteroclita* (Roxb.) Craib were collected in January 2018. Both samples were identified and authenticated by Assist. Prof. Dr. Chunthana Suwanthada and Assoc. Prof. Dr. Chusri Trisonthi of the Plant Genetic Conservation Project under the royal initiative of Her Royal Highness Princess Maha Chakri Sirindhorn. The voucher specimens, including BK No. 071406 (*Kadsura coccinea* (Lem.) A.C. Sm.) and BK No. 071407 (*Kadsura heteroclita* (Roxb.) Craib), were deposited at the Bangkok Herbarium (BK), Bangkok, Thailand.

All fresh samples were cleaned with deionized water and prepared as exocarp, mesocarp (edible part), seed, and core (Supplementary Table S1). All the samples were cut (approximately 0.3 cm thick) before freeze drying by a Heto Powerdry PL9000 freeze dryer (Heto Lab Equipment, Allerod, Denmark) for 3 days. Dry samples were then ground using a grinder (Philips 600 W series from Philips Electronic Co., Ltd., Jakarta, Indonesia) into a fine powder before packing in a vacuum aluminum foil bag and storing in a freezer at −20 °C for further analysis.

A spectrophotometer (ColorFlex EZ, Hunter Associates Laboratory, Reston, VA, USA) was used to analyze the colors of the fresh and dry samples, which were expressed as CIELAB units, where L* represents dark (0) to white (100) colors, a* represents green (−) to red (+) colors, and b* represents blue (−) to yellow (+) colors. The moisture content of the powdered samples was analyzed using a Halogen moisture analyzer (HE53 series, Mettler-Toledo AG, Greifensee, Switzerland). The color data and moisture content data are presented in Supplementary Table S2.

The extraction of *Kadsura* spp. was optimized as described by a previous study [16]. Briefly, the powered samples (100 mg) were dissolved in distilled water (10 mL) and incubated in a 90 °C temperature-controlled water bath shaker (WNE45 series from Memmert GmBh, Eagle, WI, USA) for 1 h. The supernatant was then collected by centrifugation at 3800× *g* using a Hettich^®^ ROTINA 38R refrigerated centrifuge (Andreas Hettich GmbH, Tuttlingen, Germany) for 15 min and filtered through a 0.45 µM PES membrane syringe filter. All the extracted samples were stored at −20 °C for further analysis.

### 2.2. Determination of Antioxidant Activity

The antioxidant activities, including 2,2-diphenyl-1-picrylhydrazyl (DPPH) radical scavenging, ferric ion reducing antioxidant power (FRAP), and oxygen radical absorbance capacity (ORAC) assays of the *Kadsura* spp. extracts, naringenin, and quercetin were determined using existing, well-established protocols [17,18,19,20].

The DPPH radical scavenging assay was performed using DPPH in 95% (*v*/*v*) aqueous ethanol as previously described [17,20]. Trolox solution (0.01–0.64 mM) was used as a standard, and the results were reported as µmol TE/g dried weight (DW).

The FRAP assay was determined using FRAP reagent as previously described [18,20]. Trolox solution (7.81–250.00 µM) was used as a standard, and the results were reported as µmol TE/g DW.

The ORAC assay was examined using fluorescein reagent as previously described [19,20]. Trolox solution (3.12–100.00 µM) was used as a standard, and the results were reported as µmol TE/g DW.

### 2.3. Determination of Total Phenolic Contents, Total Flavonoid Contents (TFCs), Total Anthocyanin Contents (TACs), and Phenolic Profiles

The total phenolic contents (TPCs) of the *Kadsura* spp. extracts were determined using Folin-Ciocalteu reagent as previously described [21,22]. Gallic acid (10–200 µg/mL) was used as a standard, and the TPCs were reported as the mg gallic acid equivalent (GAE)/g DW.

Total flavonoid contents (TFCs) of the *Kadsura* spp. extracts were determined using aluminum trichloride as previously described [23]. Quercetin (0–100 µg/mL) was used as a standard, and the TFCs were reported as the mg quercetin equivalent (QE)/g DW.

Total anthocyanidin contents (TACs) of the *Kadsura* spp. extracts were determined using the pH differential method as previously described [24]. Cyanidin 3-*O*-glucoside (2–63 µg/mL) was used as a standard, and the TACs were reported as the mg cyanidin 3-*O*-glucoside equivalent (C3GE)/g DW.

Phenolic profiles were analyzed using high performance liquid chromatography (HPLC) as described previously [25]. The powdered samples (0.5 g) were mixed with 62.5% (*v*/*v*) aqueous methanol containing 0.5 g/L tBHQ (40 mL) and 6 N HCl (10 mL) before incubating in a 80 °C temperature-controlled water bath shaker (WNE45 series from Memmert GmBh, Eagle, WI, USA) for 2 h in dark. The mixture was then cooled in ice for 5 min before sonicating in an ultrasonic cleansing bath (Branson Ultrasonics™ M series, Branson Ultrasonics Corp., Danbury, CT, USA) for a further 5 min. The mixture was filtered through a 0.22 µM PTFE membrane syringe filter. Phenolic acids in the filtrate were analyzed by HPLC utilizing a 5 µm Zorbax Eclipse XDB-C_18_ column (150 × 4.6 mm from Agilent Technologies, Santa Clara, CA, USA) on an Agilent 1100 HPLC system with a photodiode array detector from Agilent Technologies (Santa Clara, CA, USA). The gradient mobile phases comprised Milli-Q water (18.2 MΩ.cm resistivity at 25 °C) containing 0.05% (*v*/*v*) TFA (solvent A), methanol containing 0.05% (*v*/*v*) TFA (solvent B), and acetonitrile containing 0.05% (*v*/*v*) TFA (solvent C) with a constant flow rate of 0.6 mL/min, as shown in Table 1. The existence of the phenolic acids were visualized at 280 and 325 nm using the ChemStation software (Agilent Technologies, Santa Clara, CA, USA) by comparing retention time (*t_R_*) and spectral fingerprint with standards including 4-hydroxybenzoic acid (>99.0% GC, T), caffeic acid (>98.0% HPLC, T), chlorogenic acid (>98.0% HPLC, T), ferulic acid (>98.0% GC, T), *p*-coumaric acid (>98.0% GC, T), sinapic acid (>99.0% GC, T), syringic acid (>97.0% T) from Tokyo Chemical Industry (Tokyo, Japan), and gallic acid (97.5–102.5% T) from Sigma-Aldrich (St. Louis, MO, USA).

Flavonoid identification was performed similar to that of phenolic acids. However, the existence of flavonoids was visualized at 338 and 368 nm and evaluated by comparing *t_R_* and spectral fingerprint with standards including apigenin (>98.0% HPLC), genistein (>98.0% HPLC), hesperidin (>90.0% HPLC, T), kaempferol (>97.0% HPLC), luteolin (>98.0% HPLC), myricetin (>97.0% HPLC), naringenin (>93.0% HPLC, T), quercetin (>98.0% HPLC, E) from Tokyo Chemical Industry (Tokyo, Japan), and isorhamnetin (≥99.0% HPLC) from Extrasynthese (Genay, France).

Anthocyanidin and anthocyanin identification was performed as previously described [26,27]. Anthocyanidins were extracted using the powdered sample (500 mg) that was dispersed in 50% (*v*/*v*) aqueous methanol containing 2 N HCl (5 mL). The mixture was incubated in a 100 ± 2 °C water bath (TW20 series from Julabo GmbH, Seelbach, Germany) for 1 h before filtering through a 0.22 µM PTFE membrane syringe filter. Anthocyanidins were identified by the HPLC system (an Ultimate 3000 with diode array and multiple-wavelength detectors) from Thermo Fisher Scientific (Dreieich, Germany) and a 5 µm ReproSil-Pur^®^ ODS-3 column (250 × 4.6 mm) from Dr. Maisch GmbH (Ammerbuch, Germany). Milli-Q water (18.2 MΩ.cm resistivity) containing 0.4% (*v*/*v*) TFA (solvent A) and acetonitrile containing 0.4% *v*/*v* TFA (solvent B) were used as an isocratic mobile phase at a ratio of 82% solvent A and 18% solvent B, with a constant flow rate of 1.0 mL/min. The existence of anthocyanidins was visualized at 530 nm using a Chromeleon™ chromatography Data System (CDS) software (Thermo Fisher Scientific, Dreieich, Germany) by comparing *t_R_* and spectral fingerprint with the standards including cyanidin (≥96.0% HPLC), delphinidin (≥97.0% HPLC), pelargonidin (≥97.0% HPLC), peonidin (≥97.0% HPLC), and petunidin (≥95.0% HPLC) from Extrasynthese (Genay, France).

Anthocyanin identification was performed similarly to the anthocyanidin identification. Anthocyanins were extracted using the powdered sample (500 mg) that was dispersed in 50% (*v*/*v*) aqueous methanol containing 2% (*v*/*v*) HCl (5 mL). The extract was then sonicated using an ultrasonic cleansing bath (Branson Ultrasonics™ M series, Branson Ultrasonics Corp., Danbury, CT, USA) for 20 min and filtered through a 0.22 µM PTFE membrane syringe filter. Anthocyanins were identified utilizing the same HPLC system as anthocyanidins. Milli-Q water containing 0.4% (*v*/*v*) TFA (solvent A) and acetonitrile containing 0.4% (*v*/*v*) TFA (solvent B) were used as gradient mobile phases with a constant flow rate 1.0 mL/min, as shown in Table 2. The existence of anthocyanins was visualized at 525 nm and compared *t_R_* and spectral fingerprint with the standards including cyanidin 3-*O*-sophoroside (≥95.0% HPLC), cyanidin 3,5-di-*O*-glucoside (≥97.0% HPLC), cyanidin 3-*O*-galactoside (≥97.0% HPLC), cyanidin 3-*O*-rutinoside (≥96.0% HPLC), cyanidin 3-*O*-glucoside (≥96.0% HPLC), and delphinidin 3,5-di-*O*-glucoside (≥97.0% HPLC) from Extrasynthese (Genay, France).

### 2.4. Determination of Enzyme Inhibitory Activities

The α-glucosidase inhibitory activity was determined as previously described [28]. Briefly, the assay comprising 2 mM *p*-nitrophenyl-α-D-glucopyranoside (50 µL) in a 50 mM phosphate buffer (pH 7.0), 0.1 U/mL *Saccharomyces cerevisiae* α-glucosidase (type 1, ≥10 U/mg protein, 100 µL), and sample extract (50 µL) was visualized at a wavelength of 405 nm using a microplate reader (Synergy™ HT 96-well UV-visible spectrophotometer using the Gen5 data analysis software from BioTek Instruments, Inc., Winooski, VT, USA). The inhibition percentage was then calculated as follows:

$$\%\;\mathrm{inhibition}\;=\;\left(1-\;\frac{B-b}{A-a}\right)\;\times \;100$$

where *A* is the initial velocity of the reaction with the enzyme, *a* is the initial velocity of the reaction without the enzyme, *B* is the initial velocity of the enzyme reaction with the extract, and *b* is the initial velocity of the reaction with the extract but without the enzyme. The efficiency of flavonoids against α-glucosidase was also determined using the half maximal inhibitory concentration (IC_50_), and analyzed by a dose-response plot of flavonoids versus the inhibition percentage.

The α-amylase inhibitory activity was determined as previously described [29]. Briefly, the assay comprising of 30 mM *p*-nitrophenyl-α-D-maltohexaoside (50 µL) in a 50 mM phosphate buffer (pH 7.0) containing 200 mM KCl, 30 mg/mL of porcine pancreatic α-amylase (type VII, ≥10 unit/mg, 100 µL), and the sample extract (50 µL) was visualized at a wavelength of 405 nm using the microplate reader. The inhibition percentage was then calculated as above.

Acetylcholinesterases (AChE) inhibitory activities were determined as previously described [25]. The enzyme assay consisting of 20 ng of *Electrophorus electricus* AChE (1000 units/mg, 100 μL) in 50 mM KPB (pH 7.0), 16 mM 5,5-dithio-bis-(2-nitrobenzoic acid) (DTNB, 10 μL), 0.8 mM acetylthiocholine (40 μL) in 50 mM KPB (pH 7.0), and the extract (50 μL) was detected at a wavelength of 412 nm using the microplate reader. Butyrylcholinesterases (BChE) inhibitory activities were determined similarly to AChE. However, 100 ng equine serum BChE (≥10 units/mg protein, 100 μL) in 50 mM KPB (pH 7.0) containing 1 mM MgCl_2_ and 0.1 mM butyrylthiocholine (40 μL) in 50 mM KPB (pH 7.0) were used as the enzyme and substrate, respectively. The results were expressed as the inhibition percentage and the IC_50_ value, as mentioned above.

The beta-secretase (BACE-1) inhibitory activity was determined utilizing a BACE-1 activity detection kit (Sigma-Aldrich, St. Louis, MO, USA). The manufacturer’s instructions were followed, and the results expressed as a percentage of BACE-1 inhibition, as above.

All the enzymes, chemicals, and reagents in the enzyme inhibitory assays were purchased from Sigma-Aldrich (St. Louis, MO, USA).

### 2.5. Statistical Analysis

All experiments were carried out in triplicate (*n* = 3) and expressed as mean ± standard deviation (SD). One-way analysis of variance (ANOVA) followed by Duncan’s multiple comparison test and unpaired *t*-test were performed to determine the significant differences between values with *p* < 0.05.

## 3. Results

### 3.1. Total Phenolic Contents (TPCs), Total Flavonoid Contents (TFCs), Total Anthocyanin Contents (TACs), and Phenolic Profiles

The total phenolic contents (TPCs) of *K. coccinea* (Lem.) A.C. Sm. extracts (KCE) ranged between 0.83 and 43.61 mg gallic acid equivalent (GAE)/g dry weight (DW), while *K. heteroclita* (Roxb.) Craib extract (KHE) exhibited the TPCs ranging from 1.13 to 103.72 mg GAE/g DW (Table 3). Among the different fruit parts, the exocarp of KCE exhibited the significantly highest TPCs, followed by the core, mesocarp, and seed, respectively. However, for KHE, its core exhibited significantly higher TPCs than exocarp, mesocarp, and seed, respectively. Furthermore, significantly higher TPCs were observed in all the fruit parts of KHE compared to KCE.

Similarly, the total flavonoid contents (TFCs) in KCE ranged between 1.45 and 60.52 mg quercetin equivalent (QE)/g DW, with the exocarp providing the highest TFCs, followed by the core, while mesocarp and seed exhibited the lowest TFCs (Table 3). The core of KHE exhibited the highest TFCs, followed by the exocarp, mesocarp, and seed, respectively. In line with the TPC results, higher TFCs were detected in all the fruit parts of KHE (ranging from 1.40 to 206.92 mg QE/g DW) compared to KCE, in exception of seed.

Interestingly, the total anthocyanin contents (TACs) of KCE were only detected in the exocarp and mesocarp (0.02–0.27 mg cyanidin 3-*O*-glucoside equivalent (C3GE)/g DW), while TACs in KHE were found in the exocarp, mesocarp, and core (0.14–0.28 mg C3GE/g DW) (Table 3). The mesocarp of KCE exhibited higher TACs than its exocarp. Meanwhile, the exocarp and mesocarp of KHE exhibited insignificantly different TACs, but were significantly higher than that of the core.

Among all the phenolic acids and flavonoids investigated utilizing high performance liquid chromatography (HPLC), quercetin and naringenin were the only flavonoids identified in *Kadsura* spp. extracts (Table 4, Supplementary Figures S1 and S2). The most abundant flavonoid, naringenin, was detected in all fruit parts of KCE (ranging from 1395.22 to 1972.65 mg/100 g DW) and KHE (ranging from 1739.91 to 1835.46 mg/100 g DW). Meanwhile, quercetin was only found in the exocarp of KCE (17.94 mg/100 g DW), and in the exocarp and mesocarp of KHE (51.19 and 12.59 mg/100 g DW, respectively). Among all the fruit parts, the exocarp and seed of KCE exhibited significantly higher naringenin content than the core and mesocarp, respectively. However, insignificant naringenin content differences were detected in all the fruit parts of KHE.

Anthocyanidins detected in *Kadsura* spp. extracts were identified as cyanidin and delphinidin (Table 4 and Supplementary Figure S3). Only the exocarp, mesocarp, and core of both *Kadsura* spp. extracts were found to contain these anthocyanidins, while none was detected in the seed. KCE exhibited cyanidin contents in the range of 0.34–1.03 µg/100 g DW, with its exocarp and mesocarp containing higher cyanidin content compared to its core. Similar results were observed in KHE (cyanidin content ranging from 0.03 to 1.00 µg/100 g DW), in which its exocarp and mesocarp provided higher cyanidin contents than its core. Delphinidin in KCE ranged between 0.42 and 4.02 µg/100 g DW, while KHE exhibited delphinidin contents in the range of 0.31–0.50 µg/100 g DW. The exocarp in both *Kadsura* spp. extracts provided higher delphinidin content than the mesocarp and core, respectively.

Anthocyanins (or glycosylated anthocyanidins) detected in *Kadsura* spp. extracts were identified as cyanidin 3,5-di-*O*-glucoside (cyanin), cyanidin 3-*O*-galactoside (ideain), cyanidin 3-*O*-glucoside (kuromanin), and cyanidin 3-*O*-rutinoside (keracyanin) (Table 4 and Supplementary Figure S4). Only the mesocarp of KCE contained cyanin (9.94 µg/100 g DW) and ideain (10.16 µg/100 g DW), while none was detected in the other fruit parts. Meanwhile, KHE exhibited ideain (2.11–53.36 µg/100 g DW), kuromanin (0.27–26.06 µg/100 g DW), and keracyanin (4.16–112.54 µg/100 g DW) in the exocarp, mesocarp, and core, while none was detected in the seed. The exocarp contained higher content for all three anthocyanins compared to the mesocarp and core, respectively. Besides, kuromanin and keracyanin were only detected in KHE, while none was found in KCE. On the other hand, cyanin was only found in KCE, while none was detected in KHE.

### 3.2. Antioxidant Activities

Antioxidant activities of *Kadsura* spp. extracts were determined using 2,2-diphenyl-1-picrylhydrazyl (DPPH) radical scavenging activity, ferric ion reducing antioxidant power (FRAP), and oxygen radical absorbance capacity (ORAC) assays (Table 5). KCE exhibited DPPH radical scavenging activities in the range of 0.04–2.73 µmol trolox equivalent (TE)/g DW, FRAP activities in the range of 3.14–300.44 µmol TE/g DW, and ORAC activities in the range of 41.93–957.80 µmol TE/g DW. Among all fruit parts, the exocarp provided significantly higher antioxidant activities than the core, mesocarp, and seed, respectively. This data corresponds with the TPCs, in which the highest TPCs were detected in the exocarp of KCE and the lowest in the seed. Similarly, KHE exhibited DPPH radical scavenging activities in the range of 0.04–6.48 µmol TE/g DW, FRAP activities in the range of 2.91–900.60 µmol TE/g DW, and ORAC activities in the range of 15.84–1330.23 µmol TE/g DW. Among all fruit parts, the core exhibited significantly higher antioxidant activities, followed by the exocarp, mesocarp, and seed, respectively. This data corresponds to the TPCs and TFCs, in which the highest TPCs and TFCs were detected in the core of KHE and the lowest in the seed. Interestingly, considering the abundant flavonoids detected in *Kadsura* spp., naringenin and quercetin, the results suggested that quercetin is a stronger antioxidant with higher DPPH radical scavenging activity of 8.07 µmol TE/g DW, FRAP activity of 13,519.54 µmol TE/g DW, and ORAC activity of 30,112.47 µmol TE/g DW than naringenin with lower DPPH radical scavenging activity of 0.02 µmol TE/g DW, FRAP activity of 30.98 µmol TE/g DW, and ORAC activity of 25,592.45 µmol TE/g DW.

### 3.3. In Vitro Enzyme Inhibitory Activities

*Kadsura* spp. extracts inhibited the key enzymes relevant to diabetes, including α-glucosidase and α-amylase, with different degrees of inhibition (Table 6). The α-glucosidase inhibitory activities in both *Kadsura* spp. extracts were detected in all fruit parts except for the seed. KCE exhibited α-glucosidase inhibitory activities in the range of 60.04–98.23% inhibitions using an extract concentration of 0.5 mg/mL, while those in KHE ranged between 89.72% and 91.84% inhibitions with the same extract concentration. It is also found that the exocarp and core of both *Kadsura* spp. extracts exhibited significantly higher α-glucosidase inhibitory activities than mesocarp, as indicated by the half maximal inhibitory concentration (IC_50_) (Table 6). Lower IC_50_ values indicated greater effectiveness of enzyme inhibitions. The exocarp and core of KCE exhibited significantly lower IC_50_ values (0.13 and 0.45 mg/mL, respectively) than mesocarp (0.56 mg/mL). Similar results were observed with KHE, in which its exocarp and core exhibited significantly lower IC_50_ values (0.06 mg/mL) than its mesocarp (the IC_50_ value of 0.64 mg/mL). Interestingly, the exocarp and core of KHE exhibited significantly lower IC_50_ values compared to those of KCE, while insignificantly different IC_50_ values were observed in the mesocarp of both *Kadsura* spp. extracts.

Likewise, KCE exhibited α-amylase inhibitory activities in the range of 12.18–51.85% inhibitions using an extract concentration of 2.5 mg/mL, while those in KHE ranged between 33.93% and 44.75% inhibitions when using the same extract concentration. When comparing the different fruit parts of KCE, its exocarp exhibited significantly higher inhibitory activities than its core, mesocarp, and seed, respectively. Similar results were observed in KHE, in which its exocarp and core possessed significantly higher inhibitory activities than its mesocarp, but no inhibitory activity was observed in its seed. Due to low inhibitory activities, the IC_50_ value against α–amylase of *Kadsura* spp. extracts is unavailable.

*Kadsura* spp. extracts were able to inhibit acetylcholinesterase (AChE), butyrylcholinesterase (BChE), and beta-secretase (BACE-1), the key enzymes relevant to AD with different degrees of inhibition. The AChE inhibitory activities in KCE ranged between 20.40% and 87.44% inhibitions, while those of KHE ranged between 29.98% and 91.74% inhibitions using the extract concentration of 2.0 mg/mL. The exocarp and core of both *Kadsura* spp. extracts possessed significantly higher AChE inhibitory activities than mesocarp, while no inhibitory activities were detected in the seed. Due to the low inhibitory activities in the mesocarp, the IC_50_ values against AChE were only detected in exocarp and core. KCE exhibited the IC_50_ values of 0.88 mg/mL in the exocarp and 1.54 mg/mL in the core, while KHE exhibited the IC_50_ values of 0.52 mg/mL in the exocarp and 0.41 mg/mL in the core (Table 6). It is also suggested that the KHE exocarp and core exhibited significantly lower IC_50_ values than those of KCE.

Similar results were observed with BChE inhibition, in which only the exocarp, mesocarp, and core of both *Kadsura* spp. extracts possessed inhibitory activities, while none was detected in the seed. KCE exhibited BChE inhibitory activities in the range of 41.27–96.81% inhibitions, while those of KHE ranged between 63.67% and 99.15% inhibitions using the same extract concentration of 2.0 mg/mL. The exocarp and core of KHE exhibited significantly lower IC_50_ values (0.20 and 0.16 mg/mL, respectively) than its mesocarp (1.32 mg/mL) (Table 6). However, KCE exhibited significantly lower IC_50_ values in its exocarp (0.49 mg/mL) than its core (0.67 mg/mL). Interestingly, the exocarp and core of KHE exhibited significantly lower IC_50_ values than those of KCE.

Additionally, KCE exhibited BACE-1 inhibitory activity in the range of 4.72–56.34% inhibitions, while those of KHE ranged between 12.70% and 35.26% inhibitions using the same extract concentration of 2.0 mg/mL. In KCE, the mesocarp exhibited a significantly higher BACE-1 inhibitory activity than the exocarp, core, and seed, respectively. Similar results were observed in KHE, in which the mesocarp exhibited the significantly higher BACE-1 inhibitory activity, followed by the exocarp and seed, respectively. No inhibitory activity was observed in the core of KHE.

## 4. Discussion

Two species of *Kadsura* spp., *K. coccinea* (Lem.) A.C. Sm. and *K. heteroclita* (Roxb.) Craib, are found abundantly in Thailand. *Kadsura* spp. has been reportedly used as a folk medicine for several decades. Despite their valuable medicinal applications, *Kadsura* spp. can only be naturally populated in the forest, which makes them unrecognizable for outsiders, while knowledge of their biological properties is also limited. The present research is the first comparative and comprehensive study of two *Kadsura* spp. in terms of their phytochemicals (total phenolic contents (TPCs), total flavonoid contents (TFCs), total anthocyanin contents (TACs), and phenolic profiles), and their health properties (in vitro antioxidant, anti-diabetic, and anti-Alzheimer’s properties). The researchers found that (i) *Kadsura* spp. extracts were rich in flavonol (quercetin), flavanone (naringenin), anthocyanidins (cyanidin and delphinidin), and anthocyanins (cyanidin 3-*O*-glucoside (kuromanin), cyanidin 3-*O*-galactoside (ideain), cyanidin 3-*O*-rutinoside (keracyanin), and cyanidin 3,5-di-*O*-glucoside (cyanin)); (ii) the antioxidant activities of *Kadsura* spp. extracts were related to their phenolic contents; (iii) *Kadsura* spp. extracts exhibited strong inhibitory activities against key diabetic enzymes, including α-glucosidase and α-amylase; (iv) *Kadsura* spp. extracts provided effective in vitro anti-Alzheimer properties through acetylcholinesterase (AChE), butyrylcholinesterase (BChE), and beta-secretase (BACE-1) inhibitions. This information supports the further development of *Kadsura* spp. as a potential source of phenolics with health benefits against the occurrence of diabetes and AD.

Flavonoids are diphenylpropanes-structured phytochemicals, which are mostly found in plant-based diets. The HPLC outcomes reveal that the identified flavonol and flavanone in *Kadsura* spp. extracts were quercetin (12.59–51.19 mg/100 g DW) and naringenin (1395.22–1972.65 mg/100 g DW), respectively. Naringenin is the most abundant flavonoid in *Kadsura* spp. extracts and is also widely distributed in citrus fruits such as kumquats, grapefruits, yuzu, and pomelo [30]. Quercetin is the most common flavonol and is found ubiquitously in mulberry, apricot, apple, and onion [31]. The amounts of quercetin and naringenin in the *Kadsura* spp. extracts corresponded with the TPCs and TFCs, in which the exocarp appeared to exhibit higher TPCs and TFCs than mesocarp. Similar results were observed in *K. coccinea* collected in China, which suggested that the TPCs of exocarp was double those detected in mesocarp [7]. Besides, anthocyanidins, the pigmented water-soluble compounds widely found in red to purplish-blue colored plants, were also identified in *Kadsura* spp. extracts as cyanidin and delphinidin. This data corresponds to the anthocyanins found in most vegetables and fruits, in which cyanidin (50%) is the most abundant anthocyanidin, followed by delphinidin (12%), peonidin (12%), pelargonidin (12%), malvidin (7%), and petunidin (7%) [32]. Furthermore, glycosylated anthocyanidins, namely anthocyanins, were identified in *Kadsura* spp. extracts as kuromanin, ideain, keracyanin, and cyanin. These findings correspond to a previous study, suggesting that since cyanidin is the most abundant anthocyanidin found in plants, its glycosylated form, kuromanin, is also the most common form of glycoside derivatives in colored plants [32]. Besides *Kadsura* spp., these anthocyanins are also abundantly detected in most berries. Cyanin is mostly found in mulberries, pomegranate, and wild blackberry [26,27,33,34], while ideain is found in blueberries and cranberries [35,36]. Keracyanin is detected in mulberries, raspberries, blackcurrants, and redcurrants [26,27,35]. In addition, kuromanin is abundantly found in strawberries, mulberries, blackcurrants, and raspberries [26,27,35,37].

These flavonoids appear to be responsible for the high antioxidant activities detected in *Kadsura* spp. extracts. The results on antioxidant activities of individual flavonoid suggested that quercetin is a stronger antioxidant than naringenin. However, due to high content of detected naringenin, this flavonoid might be responsible for high antioxidant activities in *Kadsura* spp. extracts. When comparing fruit parts, the exocarp and core of *Kadsura* spp. extracts exhibited higher antioxidant activities than the mesocarp and seed, respectively. These results correspond with the previous study, finding that antioxidants detected in exocarp of *K. coccinea* collected in China were two times higher than its mesocarp [38]. Comparably, the ORAC values of our *Kadsura* spp. extracts (675–2943 µmol TE/100 g FW) are similar to those of high antioxidant containing fruits, including fuji apple (2589 µmol TE/100 g FW), gala apple (2828 µmol TE/100 g FW), apricot (1110 µmol TE/100 g FW), hass avocado (1922 µmol TE/100 g FW), grapefruits (1640 µmol TE/100 g FW), grapes (red, black, white, and green as 1018–1837 µmol TE/100 g FW), white-flesh guava (2550 µmol TE/100 g FW), lemon (1346 µmol TE/100 g FW), mangosteen (2510 µmol TE/100 g FW), oranges (2103 µmol TE/100 g FW), peach (1922 µmol TE/100 g FW), and green-cultivar pear (2201 µmol TE/100 g FW) [39]. Additionally, the antioxidant activities are related to the TPCs and TFCs. The phenolics are proven to be the strong anti-oxidative agents [40,41]. Naringenin, the most abundant flavonoid detected in *Kadsura* spp. extracts, can strongly inhibit oxidative stress [42] and induce endogenous antioxidants [43]. Besides, it can suppress lipid peroxidation in rat liver tissue induced by hydroxyl and peroxyl radicals [44]. Many previous studies report that the effectiveness of anti-oxidative agents is related to their structures. The antioxidant properties activated by flavonoids are due to the existence of functional hydroxyl moieties, for instance, an active 4′ hydroxyl moiety of naringenin is responsible for its antioxidant activity [45]. Since these phenolics are responsible for the antioxidant activities in *Kadsura* spp. extracts, the exocarp and core with higher TPCs and TFCs than the mesocarp and seed also exhibited higher antioxidant activities. This data suggests that *Kadsura* spp. extracts have a strong antioxidant potential, leading to health promoting biological effects.

Besides being rich sources of anti-oxidative agents, *Kadsura* spp. extracts also exhibit strong inhibitory activities against α-glucosidase and α-amylase, the key enzymes in controlling diabetes. These enzymes play a significant role in hydrolyzing carbohydrates (polysaccharides) into glucose subunits (monosaccharide) before being absorbed into the small intestine. Various plant extracts have been examined previously and reported to possess α-amylase and α-glucosidase inhibitions [46]. Moreover, epidemiological studies support that phenolics, mainly flavonoids and phenolic acids, contribute to the prevention of DM through the α-amylase and α-glucosidase inhibitory effects. The most abundant phenolic detected in *Kadsura* spp., naringenin, can act as a mixed, close to non-competitive inhibitor with the IC_50_ value of 75 µM against α-glucosidase [28,47]. Additionally, naringenin inhibited α-glucosidase activity in diabetic rats, resulting in delayed carbohydrate absorption and decreased postprandial blood glucose level [48]. However, its action against α-amylase is much lower than that of α-glucosidase, in which its IC_50_ value on α-amylase inhibition was found to be greater than 500 µM [47]. Since KHE contained higher quantities of phenolics than KCE, it also had higher α-amylase and α-glucosidase inhibitory activities than the latter. Besides, these phenolics were more effective against α-glucosidase than α-amylase, while *Kadsura* spp. extracts exhibited potentially higher α-glucosidase inhibitions than α-amylase inhibitions.

*Kadsura* spp. extracts also provide effective in vitro anti-AD properties through AChE, BChE, and BACE-1 inhibitions. Under normal conditions, acetylcholine (ACh), a neurotransmitter working as a molecular messenger between neurons, is hydrolyzed mainly by AChE and co-regulated by BChE. In the AD hypothesis, these neurotransmitters decrease as a result of overexpression of AChE and BChE, leading to cognitive impairment [16]. Recent AD treatments are based on increasing cerebral acetylcholine levels by cholinesterase inhibitors [49]. Synthetic drugs used widely to treat AD symptoms, such as tacrine and donepezil, and can introduce both effective outcomes and cause side effects such as hepatotoxicity and gastrointestinal complaints [20]. Thus, natural therapeutics such as plant extracts are of interest, since some plant phenolics have been proven to provide a strong contribution in the prevention of neurodegenerative diseases [50]. The major flavonoid contained in *Kadsura* spp. extract, naringenin, has been previously reported to exhibit AChE inhibitory activity with the IC_50_ value of 42.66 µM and BChE inhibitory activity with the IC_50_ value of over 100 µM [51]. It has also been suggested that naringenin could introduce neuroprotective effects in an in vivo study, in which naringenin was found to suppress the activity of AChE, resulting in elevated synaptic cholinergic neurotransmitter level, improved cognitive functions, and prevented memory extinction [52]. Another pathogenesis of AD is amyloids cascade formation. Amyloid beta-peptides (Aβ) are overproduced and gradually accumulated in the brain. This aggregation leads to amyloid plaques, which cause oxidative stress and neurotoxicity. Aβ is derived from proteolytic cleavage by endogenous BACE-1, an enzyme that is increasingly found in AD patients [53]. BACE-1 is, therefore, another key enzyme for AD progression. Naringenin was previously reported to inhibit BACE-1 activity with the IC_50_ value of 30.31 µM [51]. Additionally, an oral administration of naringenin could also improve memory deficits in an Aβ-induced mouse model of AD [54]. Thus, AChE, BChE, and BACE-1 inhibitory activities detected in *Kadsura* spp. extracts may be the result of the biological function of this flavonoid, which acts as effective enzyme inhibitor.

In conclusion, *Kadsura* spp., including *Kadsura coccinea* (Lem.) A.C. Sm. and *Kadsura heteroclita* (Roxb.) Craib, contained high phenolic and flavonoid contents with naringenin being the most abundant compound. This flavonoid was responsible for the antioxidant activities and inhibitory activities against the key enzymes controlling diabetes (α-glucosidase and α-amylase) and AD (AChE, BChE, and BACE-1). This supports their future application as potential sources of phenolics with health benefits against the occurrence of diabetes and AD. Interestingly, by comparing different fruit parts within the same *Kadsura* spp., some fruit parts (exocarp of KCE, and exocarp and core of KHE) appeared to exhibit higher TPCs, TFCs, and antioxidant activities than its edible mesocarp. Besides, the exocarp and core of both *Kadsura* spp. also exhibited greater enzyme inhibitory activities than mesocarp, suggesting the potential food application of other fruit parts rather than these being wasted. Most importantly, this information can also lead to the sustainable conservation and establish proper agricultural management of the nearly extinct *Kadsura* spp. by providing knowledge of their advantageous biological properties and potential applications.

## Figures and Tables

**Table 1:** Solvent system of the phenolic acids and flavonoids using high performance liquid chromatography (HPLC) analysis.

Time (min)	Flow Rate (mL/min)	Solvent A (%)	Solvent B (%)	Solvent C (%)
0	0.6	90	6	4
5	0.6	85	9	6
30	0.6	71	17.4	11.6
60	0.6	0	85	15
61	0.6	90	6	4
66	0.6	90	6	4

Solvent A = Milli-Q water containing 0.05% (*v*/*v*) TFA; solvent B = methanol containing 0.05% (*v*/*v*) TFA; solvent C = acetonitrile containing 0.05% (*v*/*v*) TFA.

**Table 2:** Solvent system of the anthocyanins using HPLC analysis.

Time (min)	Solvent A	Solvent B
0	88	12
6	88	12
8	85	15
25	85	15
25	88	12
30	88	12

Solvent A = Milli-Q water containing 0.4% (*v*/*v*) TFA; solvent B = acetonitrile containing 0.4% (*v*/*v*) TFA.

**Table 3:** Quantification of the total phenolic contents (TPCs), total flavonoid contents (TFCs), and total anthocyanin contents (TACs) in different fruit parts of *Kadsura coccinea* (Lem.) A.C. Sm. extract (KCE) and *Kadsura heteroclita* (Roxb.) Craib extract (KHE).

Kadsura spp.	Total Phenolic Contents (mg GAE/g DW)	Total Flavonoid Contents (mg QE/g DW)	Total Anthocyanin Contents (mg C3GE/g DW)
**KCE**			
Exocarp	43.61 ± 0.65 ^a,^*	60.52 ± 5.51 ^a,^*	0.02 ± 0.00 ^b,^*
Mesocarp	6.20 ± 0.15 ^c,^*	1.45 ± 0.52 ^c,^*	0.27 ± 0.02 ^a^
Seed	0.83 ± 0.08 ^d,^*	3.48 ± 1.06 ^c,^*	ND
Core	17.71 ± 0.07 ^b,^*	43.23 ± 1.18 ^b,^*	ND
**KHE**			
Exocarp	54.00 ± 2.11 ^B^	113.36 ± 4.87 ^B^	0.28 ± 0.02 ^A^
Mesocarp	21.20 ± 0.54 ^C^	33.02 ± 1.55 ^C^	0.26 ± 0.01 ^A^
Seed	1.13 ± 0.14 ^D^	1.40 ± 0.10 ^D^	ND
Core	103.72 ± 1.14 ^A^	206.92 ± 4.08 ^A^	0.14 ± 0.01 ^B^

All data are expressed as mean ± standard deviation (SD) of triplicate experiments (*n* = 3). GAE: gallic acid equivalent; QE: quercetin equivalent; C3GE: cyanidin 3-*O*-glucoside equivalent; DW: dry weight; ND: not detected. Lower case and upper case letters indicate significant differences (*p* < 0.05) in different fruit parts of KCE and KHE, respectively, using one-way analysis of variance (ANOVA) and Duncan’s multiple comparison test; * showed significant difference (*p* < 0.05) in the same fruit part when comparing between KCE and KHE using the unpaired *t*-test.

**Table 4:** Phenolic profiles in different fruit parts of *Kadsura coccinea* (Lem.) A.C. Sm. extract (KCE) and *Kadsura heteroclita* (Roxb.) Craib extract (KHE).

Kadsura spp.	Flavonoids (mg/100 g DW)		Anthocyanidins (µg/100 g DW)		Anthocyanins (µg/100 g DW)			
	Quercetin	Naringenin	Cyanidin	Delphinidin	Cyanin	Ideain	Kuromanin	Keracyanin
**KCE**								
Exocarp	17.94 ± 0.96 *	1972.65 ± 135.07 ^a^	1.03 ± 0.13 ^a,^*	4.02 ± 0.91 ^a^	ND	ND	ND	ND
Mesocarp	ND	1395.22 ± 50.23 ^c,^*	0.99 ± 0.38 ^b^	0.42 ± 0.19 ^a^	9.94 ± 0.20	10.16 ± 0.19 *	ND	ND
Seed	ND	1861.15 ± 31.35 ^ab^	ND	ND	ND	ND	ND	ND
Core	ND	1752.54 ± 0.70 ^b^	0.34 ± 0.05 ^b^	0.60 ± 0.29 ^b,^*	ND	ND	ND	ND
**KHE**								
Exocarp	51.19 ± 1.92 ^A^	1811.02 ± 15.28 ^A^	1.00 ± 0.07 ^A^	0.50 ± 0.02 ^A^	ND	53.36 ± 2.40 ^A^	26.06 ± 4.08 ^A^	112.54 ± 9.65 ^A^
Mesocarp	12.59 ± 1.17 ^B^	1812.07 ± 88.25 ^A^	0.41 ± 0.07 ^B^	0.43 ± 0.11 ^AB^	ND	6.83 ± 0.49 ^B^	2.68 ± 0.98 ^B^	12.56 ± 0.65 ^B^
Seed	ND	1835.46 ± 54.82 ^A^	ND	ND	ND	ND	ND	ND
Core	ND	1739.91 ± 55.90 ^A^	0.03 ± 0.01 ^C^	0.31 ± 0.09 ^B^	ND	2.11 ± 0.08 ^C^	0.27 ± 0.07 ^B^	4.16 ± 0.56 ^B^

All data are expressed as mean ± standard deviation (SD) of triplicate experiments (*n* = 3). Cyanidin 3,5-di-*O*-glucoside: cyanin; cyanidin 3-*O*-galactoside: ideain; cyanidin 3-*O*-glucoside: kuromanin; cyanidin 3-*O*-rutinoside: keracyanin; DW: dry weight; ND: not detected. Lower case and upper case letters indicate significant differences (*p* < 0.05) in different fruit parts of KCE and KHE, respectively, using one-way analysis of variance (ANOVA) and Duncan’s multiple comparison test; * showed significant difference (*p* < 0.05) in the same fruit part when comparing between KCE and KHE using unpaired *t*-test.

**Table 5:** Antioxidant activities in the different fruit parts of *Kadsura coccinea* (Lem.) A.C. Sm. extract (KCE) and *Kadsura heteroclita* (Roxb.) Craib extract (KHE) and their detected flavonoids, naringenin and quercetin.

Kadsura spp.	Antioxidant Activities (µmol TE/g DW)		
	DPPH Radical Scavenging Assay	FRAP Assay	ORAC Assay
**KCE**			
Exocarp	2.73 ± 0.08 ^a^	300.44 ± 12.09 ^a,^*	957.80 ± 77.76 ^a,^*
Mesocarp	0.23 ± 0.00 ^c,^*	25.80 ± 1.04 ^c,^*	120.20 ± 10.88 ^c,^*
Seed	0.04 ± 0.00 ^d^	3.14 ± 0.18 ^d,^*	41.93 ± 4.17 ^d,^*
Core	1.00 ± 0.03 ^b,^*	100.19 ± 1.69 ^b,^*	440.35 ± 33.60 ^b,^*
**KHE**			
Exocarp	2.75 ± 0.10 ^B^	351.48 ± 18.79 ^B^	812.66 ± 77.67 ^B^
Mesocarp	1.02 ± 0.03 ^C^	143.23 ± 12.29 ^C^	260.91 ± 23.48 ^C^
Seed	0.04 ± 0.00 ^D^	2.91 ± 0.24 ^D^	15.84 ± 1.41 ^D^
Core	6.48 ± 0.22 ^A^	900.60 ± 6.22 ^A^	1330.23 ± 49.67 ^A^
Flavonoids			
Naringenin	0.02 ± 0.00	30.98 ± 0.69	25,592.45 ± 495.92
Quercetin	8.07 ± 0.25	13,519.54 ± 242.20	30,112.47 ± 1631.24

All data are expressed as mean ± standard deviation (SD) of triplicate experiments (*n* = 3). TE: trolox equivalent; DW: dry weight. Lower case and upper case letters indicate significant differences (*p* < 0.05) in different fruit parts of KCE and KHE, respectively, using one-way analysis of variance (ANOVA) and Duncan’s multiple comparison test; * showed significant difference (*p* < 0.05) in the same fruit part when comparing between KCE and KHE using unpaired *t*-test.

**Table 6:** In vitro enzyme inhibitory activities in different fruit parts of *Kadsura coccinea* (Lem.) A.C. Sm. extract (KCE) and *Kadsura heteroclita* (Roxb.) Craib extract (KHE).

Kadsura spp.	α-Glucosidase		α-Amylase	AChE		BChE		BACE1
	%Inhibition ^1^	IC_50_ (mg/mL)	%Inhibition ^2^	%Inhibition ^3^	IC_50_ (mg/mL)	%Inhibition ^3^	IC_50_ (mg/mL)	%Inhibition ^3^
**KCE**								
Exocarp	92.32 ± 7.04 ^a^	0.13 ± 0.01 ^a,^*	51.58 ± 3.52 ^a,^*	87.44 ± 8.67 ^a^	0.88 ± 0.01 ^a,^*	96.81 ± 1.26 ^a,^*	0.49 ± 0.02 ^a,^*	41.39 ± 2.56 ^b,^*
Mesocarp	60.04 ± 5.17 ^b,^*	0.56 ± 0.03 ^c^	40.17 ± 3.31 ^c,^*	20.40 ± 1.60 ^c,^*	N/A	41.27 ± 1.97 ^c,^*	N/A	56.34 ± 2.12 ^a,^*
Seed	ND	N/A	12.18 ± 0.71 ^d^	ND	N/A	ND	N/A	4.72 ± 2.85 ^d,^*
Core	98.23 ± 1.49 ^a,^*	0.45 ± 0.00 ^b,^*	44.78 ± 2.72 ^b^	62.86 ± 3.21 ^b,^*	1.54 ± 0.10 ^b,^*	88.86 ± 0.65 ^b,^*	0.67 ± 0.04 ^b,^*	26.36 ± 1.59 ^c^
**KHE**								
Exocarp	91.84 ± 3.76 ^A^	0.06 ± 0.01 ^A^	44.75 ± 3.52 ^A^	88.64 ± 2.34 ^A^	0.52 ± 0.05 ^A^	99.15 ± 1.53 ^A^	0.20 ± 0.04 ^A^	16.29 ± 1.16 ^B^
Mesocarp	90.11 ± 2.60 ^A^	0.64 ± 0.03 ^B^	33.93 ± 2.00 ^B^	29.98 ± 3.01 ^B^	N/A	63.67 ± 5.56 ^B^	1.32 ± 0.08 ^B^	35.26 ± 2.61 ^A^
Seed	ND	N/A	ND	ND	N/A	ND	N/A	12.70 ± 0.93 ^C^
Core	89.72 ± 3.68 ^A^	0.06 ± 0.00 ^A^	44.12 ± 3.20 ^A^	91.74 ± 0.71 ^A^	0.41 ± 0.00 ^A^	97.99 ± 0.22 ^A^	0.16 ± 0.00 ^A^	ND

All data are expressed as mean ± standard deviation (SD) of triplicate experiments (*n* = 3). ^1^ Extract concentration = 0.5 mg/mL; ^2^ Extract concentration = 2.5 mg/mL; ^3^ Extract concentration = 2.0 mg/mL; N/A: not available; ND: not detected. The extract concentrations in each enzyme assay was chosen in attempt to differentiate the inhibitory activities among different fruit parts. Lower case and upper case letters indicate significant differences (*p* < 0.05) in different fruit parts of KCE and KHE, respectively, using one-way analysis of variance (ANOVA) and Duncan’s multiple comparison test; * showed significant difference (*p* < 0.05) in the same fruit part when comparing between KCE and KHE using unpaired *t*-test.

## Supplementary Materials

The following are available online at https://www.mdpi.com/2304-8158/9/9/1222/s1, Table S1: Images of whole fruit, sectioned fruit, exocarp, mesocarp (edible part), seed, and core of *Kadsura coccinea* (Lem.) A.C. Sm. and *Kadsura heteroclita* (Roxb.) Craib; Table S2: Color (where L* describes darkness (−) to lightness (+), a* describes green (−) to red (+) colors, and b* describes indigo (−) to yellow (+)) and the percentage (%) of moisture content of fresh and freeze-dried *Kadsura* spp. Samples; Figure S1: High-performance liquid chromatograms of (A.) naringenin and *Kadsura* spp. samples including (B.) exocarp, (C.) mesocarp, (D.) seed, and (E.) core of *Kadsura coccinea* (Lem.) A.C. Sm. and (F.) exocarp, (G.) mesocarp, (H.) seed, and (I.) core of *Kadsura heteroclita* (Roxb.) Craib. Retention times (*R_t_*) of phenolics in *Kadsura* spp. extracts are indicated at a wavelength of 280 nm; Figure S2: High-performance liquid chromatograms of (A.) quercetin and *Kadsura* spp. samples including (B.) exocarp, (C.) mesocarp, (D.) seed, and (E.) core of *Kadsura coccinea* (Lem.) A.C. Sm. and (F.) exocarp, (G.) mesocarp, (H.) seed, and (I.) core of *Kadsura heteroclita* (Roxb.) Craib. Retention times (*R_t_*) of phenolics in *Kadsura* spp. extracts are indicated at a wavelength of 368 nm; Figure S3: High-performance liquid chromatograms of (A.) cyanidin, (B.) delphinidin, and *Kadsura* spp. samples including (C.) exocarp, (D.) mesocarp, (E.) seed, and (F.) core of *Kadsura coccinea* (Lem.) A.C. Sm. and (G.) exocarp, (H.) mesocarp, (I.) seed, and (J.) core of *Kadsura heteroclita* (Roxb.) Craib. Retention times (*R_t_*) of phenolics in *Kadsura* spp. extracts are indicated at a wavelength of 530 nm; Figure S4: High-performance liquid chromatograms of (A.) cyanidin 3,5-di-*O*-glucoside (cyanin), (B.) cyanidin 3-*O*-glucoside (kuromanin), (C.) cyanidin 3-*O*-galactoside (ideain), (D.) cyanidin 3-*O*-rutinoside (keracyanin), and *Kadsura* spp. samples including (E.) exocarp, (F.) mesocarp, (G.) seed, and (H.) core of *Kadsura coccinea* (Lem.) A.C. Sm. and (I.) exocarp, (J.) mesocarp, (K.) seed, and (L.) core of *Kadsura heteroclita* (Roxb.) Craib. Retention times (*R_t_*) of phenolics in *Kadsura* spp. are indicated at a wavelength of 525 nm.
