# Supplementary material for: Phenolic Profiles, Antioxidant, and Inhibitory Activities of Kadsura heteroclita (Roxb.) Craib and Kadsura coccinea (Lem.) A.C. Sm
Source: Foods. 2020 Sep 2;9(9):1222. doi: 10.3390/foods9091222 (PMC7555767; doi:10.3390/foods9091222)

Supplementary materials

Phenolic Profiles, Antioxidant, and Inhibitory Activities of *Kadsura heteroclita* (Roxb.) Craib and *Kadsura coccinea* (Lem.) A.C. Sm.

Varittha Sritalahareuthai [1], Piya Temviriyanukul [1,2], Nattira On-nom [1,2], Somsri Charoenkiatkul [1], and Uthaiwan Suttisansanee [1,2,*]

[1] Institute of Nutrition, Mahidol University, Salaya, Phuttamonthon, Nakhon Pathom 73170, Thailand; varittha.sri@hotmail.com (V.S.); piya.tem@mahidol.ac.th (P.T.); nattira.onn@mahidol.ac.th (N.O.-n.); somsri.chr@mahidol.ac.th (S.C.); uthaiwan.sut@mahidol.ac.th (U.S.)

[2] Food and Nutrition Academic and Research Cluster, Institute of Nutrition, Mahidol University, Salaya, Phuttamonthon, Nakhon Pathom 73170, Thailand

* Correspondence: uthaiwan.sut@mahidol.ac.th; Tel.: +66-(0)2800-2380 (ext. 422)

Supplementary Table S1:

Images of whole fruit, sectioned fruit, exocarp, mesocarp (edible part), seed and core of *Kadsura coccinea* (Lem.) A.C. Sm. and *Kadsura heteroclita* (Roxb.) Craib.

Fruit parts	Physical appearance	
	**Kadsura coccinea (Lem.) A.C. Sm.**	**Kadsura heteroclita (Roxb.) Craib.**
**Whole fruit**	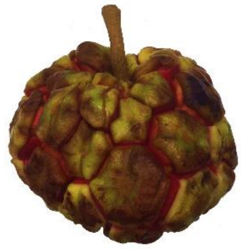	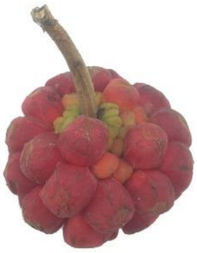
**Sectioned fruit**	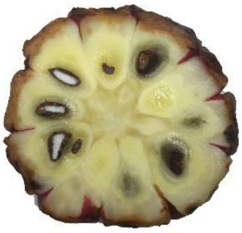	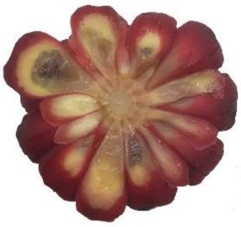
**Exocarp**	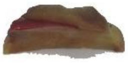	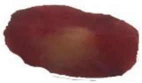
**Mesocarp**	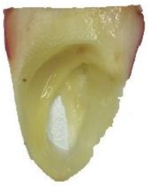	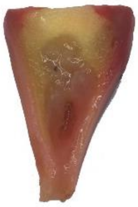
**Seed**	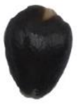	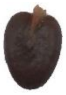
**Core**	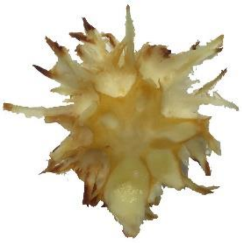	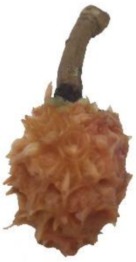

The scale (—) indicated the size of 1 cm.

Supplementary Table S2:

Color (where L* describes darkness (−) to lightness (+), a* describes green (−) to red (+) colors, and b* describes indigo (−) to yellow (+)) and the percentage (%) of moisture content of fresh and freeze-dried *Kadsura* spp. samples.

***Kadsura* spp.**	**Color of fresh samples**			**Color of dried samples**			**Moisture content (%) of fresh sample**	**Moisture content (%) of dried sample**
	**L***	**a***	**b***	**L***	**a***	**b***		
***Kadsura coccinea* (Lem.) A.C. Sm.**								
Exocarp	33.93±2.45	3.90±0.84	25.30±1.20	65.78±0.59	6.16±0.31	24.46±1.36	90.77±0.24	5.91±0.13
Mesocarp	42.76±1.33	29.17±4.18	19.41±1.32	72.45±0.83	6.75±0.49	14.13±0.39	91.46±0.23	6.24±0.44
Seed	21.17±0.66	6.59±0.28	8.64±0.78	14.73±0.26	2.74±0.10	4.17±0.11	53.56±1.00	5.15±0.05
Core	37.51±0.12	15.98±0.36	33.81±0.30	75.69±0.06	6.28±0.08	18.81±0.26	89.80±0.44	6.31±0.44
***Kadsura heteroclita* (Roxb.) Craib**								
Exocarp	34.05±10.35	15.72±3.53	16.30±2.78	39.24±5.06	12.74±3.76	9.04±4.47	88.63±0.30	6.27±0.36
Mesocarp	33.17±3.93	27.88±3.45	13.97±2.15	45.68±0.91	8.69±0.69	10.99±0.39	89.98±0.25	5.36±0.60
Seed	28.27±2.39	12.48±0.40	15.73±1.22	16.14±0.50	4.99±0.04	6.47±0.26	46.36±1.39	12.24±0.35
Core	46.39±0.55	11.38±0.27	19.48±0.19	45.98±0.01	7.74±0.01	14.99±0.02	87.79±0.29	6.76±0.29

All data were expressed as mean ± standard deviation (SD) of triplicate experiments (n = 3).

Supplementary Figure S1:

High-performance liquid chromatograms of (A.) naringenin and *Kadsura* spp. samples including (B.) exocarp, (C.) mesocarp, (D.) seed and (E.) core of *Kadsura coccinea* (Lem.) A.C. Sm. and (F.) exocarp, (G.) mesocarp, (H.) seed and (I.) core of *Kadsura heteroclita* (Roxb.) Craib. Retention times (R_t) of phenolics in *Kadsura* spp. extracts are indicated at a wavelength of 280 nm.

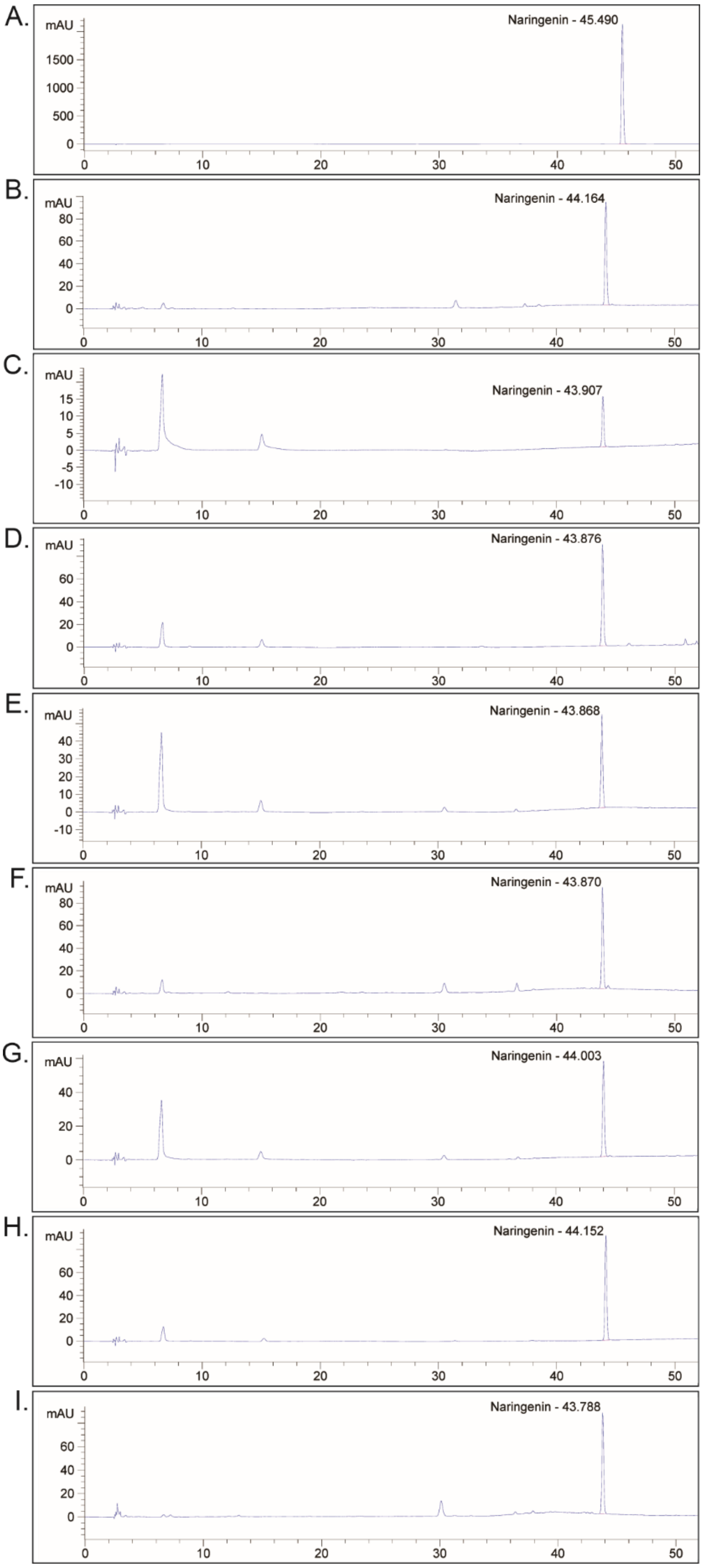

Supplementary Figure S2:

High-performance liquid chromatograms of (A.) quercetin and *Kadsura* spp. samples including (B.) exocarp, (C.) mesocarp, (D.) seed and (E.) core of *Kadsura coccinea* (Lem.) A.C. Sm. and (F.) exocarp, (G.) mesocarp, (H.) seed and (I.) core of *Kadsura heteroclita* (Roxb.) Craib. Retention times (R_t) of phenolics in *Kadsura* spp. extracts are indicated at a wavelength of 368 nm.

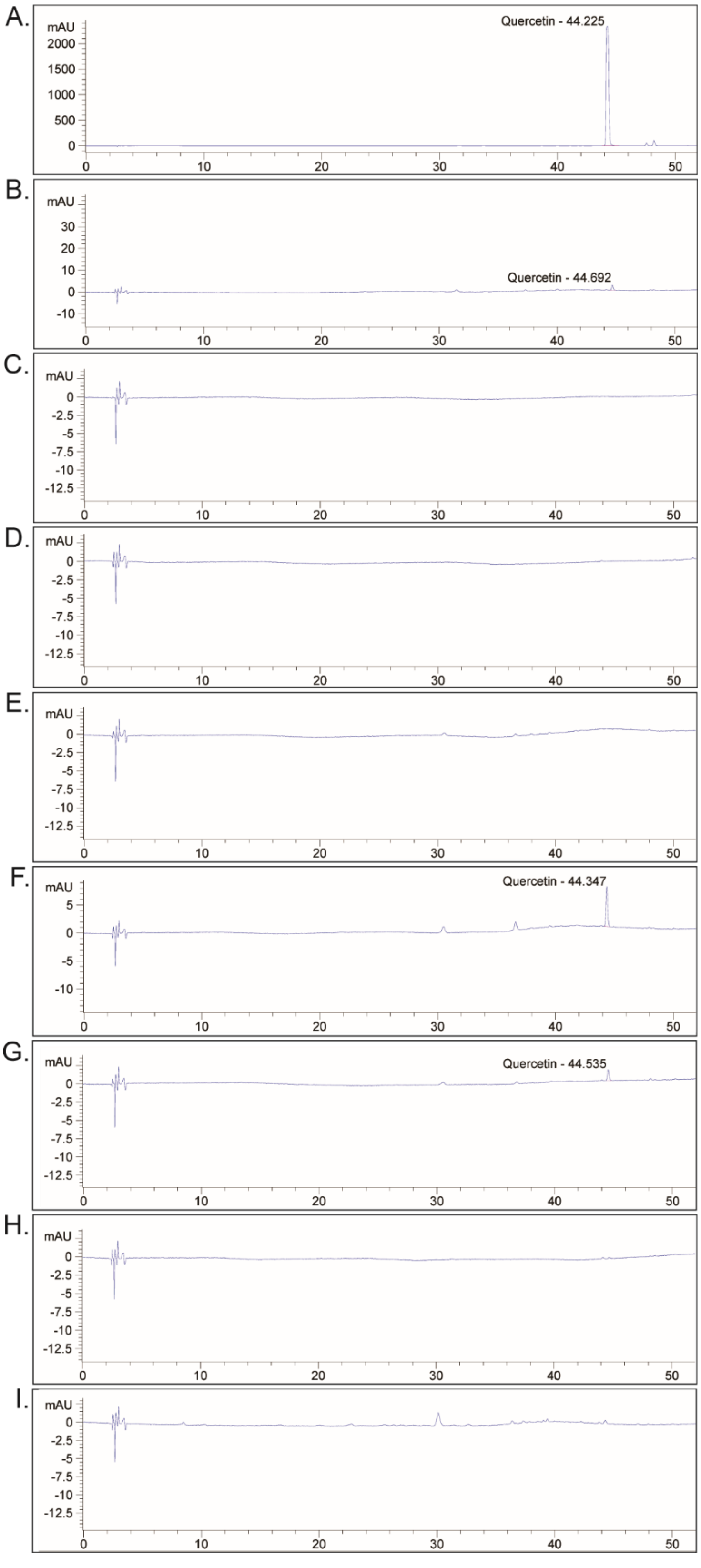

Supplementary Figure S3:

High-performance liquid chromatograms of (A.) cyanidin, (B.) delphinidin, and *Kadsura* spp. samples including (C.) exocarp, (D.) mesocarp, (E.) seed and (F.) core of *Kadsura coccinea* (Lem.) A.C. Sm. and (G.) exocarp, (H.) mesocarp, (I.) seed and (J.) core of *Kadsura heteroclita* (Roxb.) Craib. Retention times (R_t) of phenolics in *Kadsura* spp. extracts are indicated at a wavelength of 530 nm.

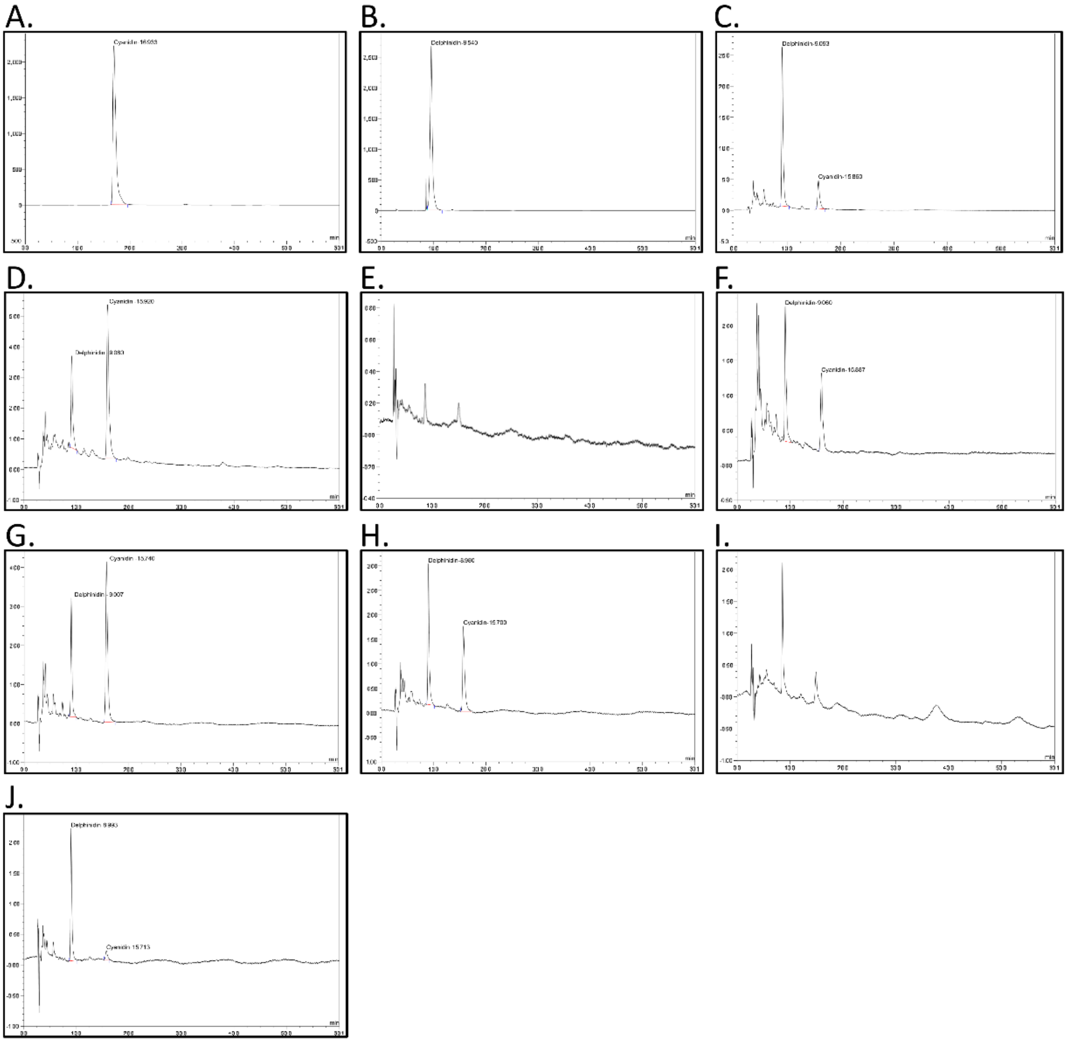

Supplementary Figure S4:

High-performance liquid chromatograms of (A.) cyanidin 3,5-di-*O*-glucoside (cyanin), (B.) cyanidin 3-*O*-glucoside (kuromanin), (C.) cyanidin 3-*O*-galactoside (ideain), (D.) cyanidin 3-*O*-rutinoside (keracyanin), and *Kadsura* spp. samples including (E.) exocarp, (F.) mesocarp, (G.) seed and (H.) core of *Kadsura coccinea* (Lem.) A.C. Sm. and (I.) exocarp, (J.) mesocarp, (K.) seed and (L.) core of *Kadsura heteroclita* (Roxb.) Craib. Retention times (R_t) of phenolics in *Kadsura* spp. are indicated at a wavelength of 525 nm.

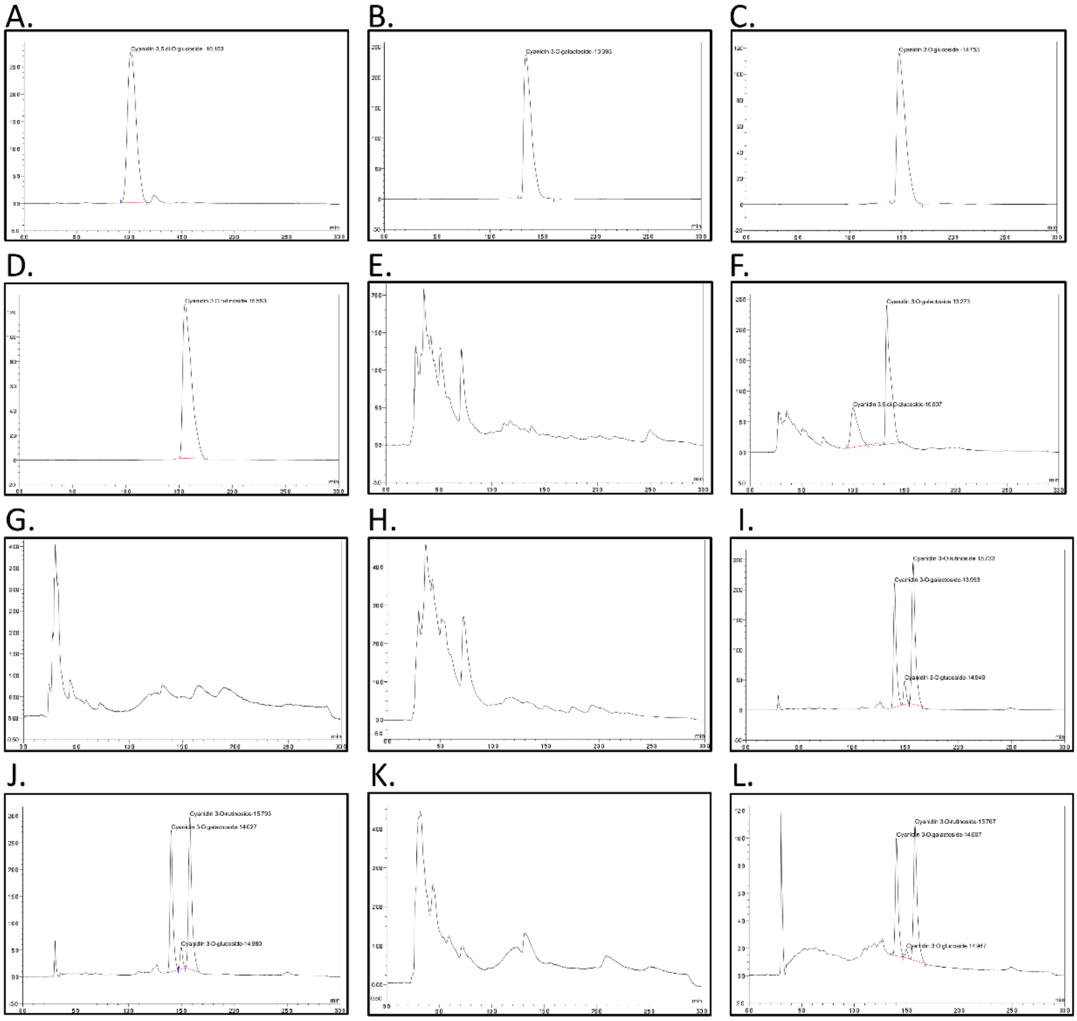

Supplement: Supplementary file 1 [file foods-09-01222-s001.pdf]
